# The Enhanced Intramolecular Energy Transfer and Strengthened ff Luminescence of a Stable Helical Eu Complex in Ionic Liquids

**DOI:** 10.3390/molecules23020055

**Published:** 2018-01-24

**Authors:** Yuki Hasegawa, Ayumi Ishii, Yudai Inazuka, Naho Yajima, Shogo Kawaguchi, Kunihisa Sugimoto, Miki Hasegawa

**Affiliations:** 1 Department of Chemistry and Biological Science, College of Science and Engineering, Aoyama Gakuin University, 5-10-1 Fuchinobe, Chuo-ku, Sagamihara 252-5258, Kanagawa, Japan; y.hasegawa106@gmail.com (Y.H.); zoo-injpn@outlook.com (Y.I.); nahoyn@gmail.com (N.Y.); 2 JST, PRESTO, 4-1-8 Honcho, Kawaguchi 332-0012, Saitama, Japan; 3 Research & Utilization Division, Japan Synchrotron Radiation Research Institute (JASRI/SPring-8), 1-1-1 Kouto, Sayo 679-5198, Hyogo, Japan; kawaguchi@spring8.or.jp (S.K.); ksugimoto@spring8.or.jp (K.S.)

**Keywords:** Eu complex, ionic liquid, luminescence, energy transfer, molecular fluctuation

## Abstract

The luminescence of a Eu complex (EuL) is enhanced by stabilization of the coordination structure in highly viscous ionic liquids. The EuL was found to maintain a stable single helical structure both in organic solvents and in the ionic liquids [BMIM][PF_6_] and [EMIM][PF_6_]. A colorless solution of EuL dissolved in [BMIM][PF_6_] exhibits bright red luminescence with a quantum yield of 32.3%, a value that is much higher than that in acetonitrile (12%). Estimated rate constants for the energy relaxation pathway indicate that the energy transfer efficiency is enhanced in [BMIM][PF_6_] as a result of the suppression of molecular fluctuations in the ligands. Additionally, a highly luminescent helical structure is preserved in [EMIM][PF_6_] up to 120 °C.

## 1. Introduction

Lanthanide (Ln) luminescence originating from electric dipole forbidden ff transitions can be sensitized through intramolecular energy transfer from photo-excited coordinated organic ligands [1,2]. These luminescence properties have received much attention because of their potential in a broad range of applications. The luminescence bands of Ln complexes appear at almost the same positions as those of other coordination compounds, although the luminescence is readily deactivated by molecular vibrations of the ligands or solvent. Therefore, obtaining highly luminescent Ln compounds requires the appropriate design of the organic ligands or the selection of an optimal media [3].

Previously, we have reported a luminescent Ln complex (LnL) having a hexadentate ligand composed of two bipyridine moieties bridged by an ethylenediamine group, which surrounds the center metal in a helical manner. Remarkably, a series of these LnL complexes exhibited high stability, maintaining a single helical molecular structure even in organic solvents such as acetonitrile [4]. EuL produces bright luminescence with a quantum yield of over 50% in the solid state, while the luminescence becomes weakened in acetonitrile (12%) because non-radiative deactivation process is enhanced by molecular fluctuations, especially in the ligand that acts as an energy donor. Very recently, the Hatanaka group determined the reason for this decreased luminescence, based on computational studies of Tb complexes with these same ligands and their derivatives [5]. They demonstrated that the single bond around the bridging and azomethine moieties is drastically transformed in the excited triplet state. These molecular fluctuations appear to be depressed in a high viscosity matrix.

In the present work, we focused on the use of ionic liquids (ILs) as a matrix for luminescent Ln complexes because ILs have been shown to act as unique donor/acceptor solvents [6]. To date, ILs have been used as alternatives for organic solvents in chemical reactions [7,8,9,10], extractions [11,12,13], and spectroscopic studies [14,15,16,17] because they have a number of useful properties, including low volatility, nonflammability, high ionic conductivity, and wide electrochemical windows [18,19]. As an example, employing ILs as solvents for spectroscopic studies results in high luminescence quantum yields from Ln ions, in addition to enhanced photostability [20]. In the case of Ln complexes, structural stability is an important aspect of maintaining the strong luminescence of the Ln ions. Thus, a stable Ln complex in an IL matrix could enhance the luminescent properties of the Ln ions.

**Figure 1:**
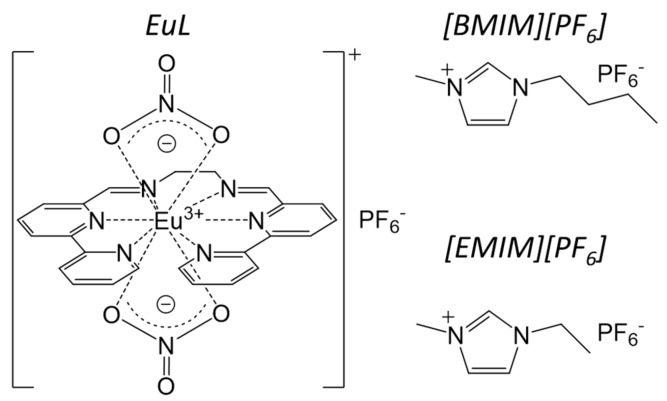
Molecular structures of the Eu complex (EuL) and the ionic liquids [BMIM][PF_6_] and [EMIM][PF_6_].

The goal of this investigation was therefore to enhance Ln luminescence by stabilization of the coordination structure in ILs. Two ILs shown in Figure 1 were selected for use with a luminescent helical Eu complex (abbreviated as EuL): 1-butyl-3-methylimidazolium hexafluorophosphate ([BMIM][PF_6_]), which has a melting point of approximately 10 °C and thus is a liquid at room temperature [21], and 1-ethyl-3-methylimidazolium hexafluorophosphate ([EMIM][PF_6_]), which has a melting point of 65 °C and thus is a solid [22]. Since both ILs have the same counter anion as the EuL, the complex is readily dissolved in these materials. Herein, we report the enhanced emission of EuL in ILs and discuss the associated mechanism based on considering molecular fluctuations, especially those of the ligand that acts as an energy donor.

## 2. Results and Discussion

### 2.1. Electronic Structure of EuL in [BMIM][PF_6_]

#### 2.1.1. Electronic Absorption and Luminescence Properties

**Figure 2:**
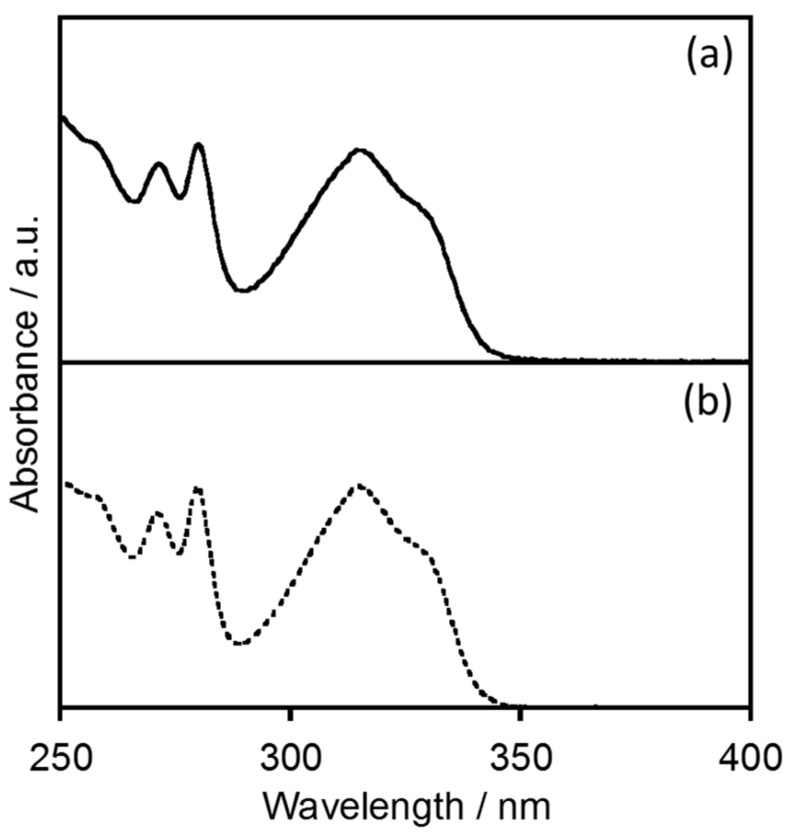
Electronic absorption spectra of the helical Eu complex with L (**a**) in [BMIM][PF_6_] compared with (**b**) in acetonitrile [4].

**Figure 3:**
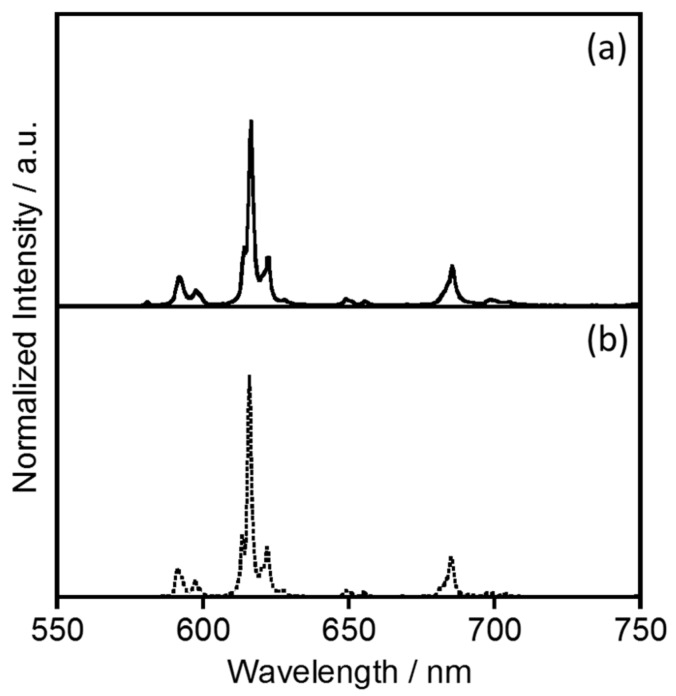
Emission spectra of the helical Eu complex with L (**a**) in [BMIM][PF_6_] compared with (**b**) in acetonitrile [4] (*λ*_ex_ = 328 nm).

Figure 2 shows the electronic absorption spectrum of EuL in [BMIM][PF_6_], compared with that in acetonitrile [4]. In [BMIM][PF_6_], the EuL generates absorption bands at 271, 280, 315, and 328 nm, assigned to the ππ* and nπ* transitions of the ligand. The spectral sharp and position observed in [BMIM][PF_6_] are almost consistent with in acetonitrile. The luminescence spectra of EuL in [BMIM][PF_6_] and acetonitrile are provided in Figure 3. The luminescence bands of EuL in [BMIM][PF_6_] are also observed at the same position as in acetonitrile. The luminescence bands at 580, 592, 616, 649, and 686 nm are assigned to the ^5^D_0_ → ^7^F_0_, ^5^D_0_ → ^7^F_1_, ^5^D_0_ → ^7^F_2_, ^5^D_0_ → ^7^F_3_, and ^5^D_0_ → ^7^F_4_ transitions of Eu^3+^, respectively (*λ*_ex_ = 328 nm). These spectral data indicate that the coordination structure of EuL in acetonitrile is maintained in [BMIM][PF_6_]. This is supported by the luminescence lifetime (*τ*_obs_) of EuL in [BMIM][PF_6_], which is almost the same as in acetonitrile (Table 1 and Figure S1.)

The excitation spectra monitored at these emission bands (Figure S2) correspond to the absorption bands positions of the ligand in Figure 1, demonstrating that intramolecular energy transfer from the ligand to Eu^3+^ takes place in [BMIM][PF_6_]. Interestingly, the luminesce quantum yield (*ϕ*_ff_) of EuL in [BMIM][PF_6_] is much higher than that in acetonitrile (Table 1). Thus, energy transfer from the ligand to Eu^3+^ evidently proceeds much more efficiently in [BMIM][PF_6_] than in acetonitrile.

#### 2.1.2. Energy Transfer Efficiency

The luminescence efficiency [1] of Eu^3+^ sensitized by the ligand (*ϕ*_ff_) is determined by the triplet yield of the ligand (*ϕ*_ISC_), the efficiency of the energy transfer (*η*_EnT_), and the efficiency of the metal-centered luminescence (*η*_Ln_), as follows:

(1) $$\phi_{\mathrm{ff}}=\;\phi_{\mathrm{ISC}}\times \;\eta_{\mathrm{EnT}}\times \;\phi_{\mathrm{Ln}}.$$

Based on the nπ* character of the ligand and the high spin-orbit coupling constants of the lanthanide ion, a *ϕ*_ISC_ value of approximately 1 can be assumed. The value of *ϕ*_Ln_ can then be calculated from the experimentally observed emission lifetime (*τ*_ff_) and the radiative rate constant (*k*_R_) of the lanthanide ion, as shown below:

(2) $$\phi_{\mathrm{Ln}}=kR\times \tau_{\mathrm{ff}}.$$

The *k*_R_ value of the emissive excited state, ^5^D_0_, is the sum of the spontaneous emission probabilities, *A_J_*(0, *J*), to the lower ^7^F*_J_* levels in Eu^3+^, and can in turn be calculated using the following equation:

(3) $$kR=\sum A_{J}(0,J)=A_{\mathrm{MD}}\times \sum A_{\mathrm{ED}}.$$

Here, the spontaneous emission probability of the magnetic dipole ^5^D_0_ → ^7^F_1_ transition, *A*_MD_, is virtually independent of the ligand field or the environment of the ions, and can be determined directly from the theoretically calculated dipole strength as follows:

(4) $$A_{\mathrm{MD}}=\frac{64\pi^{4}\sigma_{MD}^{3}n^{3}}{3h(2J+1)}\times S_{\mathrm{MD}}.$$

In the above, *σ_MD_* is the energy gap between the excited (^5^D_0_) and final (^7^F_1_) states (with a value of 16,949.2 cm^−1^), *n* is the refractive index (1.41 for [BMIM][PF_6_] and 1.34 for acetonitrile [23,24]), and *S*_MD_ is the magnetic dipole strength. The latter parameter has been calculated theoretically for the ^5^D_0_ → ^7^F_1_ transition of Eu^3+^ and found to have a value of 9.60 × 10^−42^ esu^2^ cm^2^, resulting in a value of 37.93 s^−1^ for *A*_MD_.

*A*_ED_ in Equation (3) is the emission probability associated with the electric dipole transition, as determined using the Judd–Ofelt parameter (Ω*_λ_*) in the equation below:

(5) $$A_{\mathrm{ED}}=\frac{64\pi^{4}e^{2}\sigma_{J}^{3}}{3h(2J+1)}\frac{n{\left(n^{2}+2\right)}^{2}}{9}\times \sum_{\lambda =2,4}\Omega_{\lambda }{\left|\langle \psi J\left\|U^{\left(\lambda \right)}\right\|\psi \prime J\prime \rangle \right|}^{2}.$$

Ω*_λ_* is, in turn, calculated from the following equation:

(6) $$\Omega_{\lambda }=\frac{S_{MD}}{e^{2}}\frac{\sigma_{MD}^{3}}{\sigma_{J}^{3}}\frac{9n^{2}}{{\left(n^{2}+2\right)}^{2}{\left|\langle \psi J\left\|U^{\left(\lambda \right)}\right\|\psi \prime J\prime \rangle \right|}^{2}}\frac{\int I_{J}(\nu )d\nu }{\int I_{MD}(\nu )d\nu }.$$

Here, ʃ *I*_J_ *dν*/ ʃ *I*_MD_ *dν* is the ratio of the integrated intensity of the corrected Eu^3+^ luminescence spectrum (^5^D_0_ → ^7^F*_J_*: *J* = 0, 1, 2, 3, and 4) to the intensity of the ^5^D_0_ → ^7^F_1_ band, and |〈*ΨJ*‖U^(*λ*)^ ‖*Ψ′J′*〉| is the tensor operator [25]. The Ω_2_ and Ω_4_ values are greatly affected by the ligand field symmetry around the Eu^3+^ ion, and the values for EuL in [BMIM][PF_6_] are estimated as 7.03 × 10^−20^ and 5.80 × 10^−20^ cm^2^, respectively. These values are almost the same as in acetonitrile (Ω_2_ = 7.82 × 10^−20^ cm^2^ Ω_4_ = 5.99 × 10^−20^ cm^2^), indicating that the ligand field symmetry of EuL is maintained in [BMIM][PF_6_]. Based on the Judd–Ofelt analysis, we can estimate *A*_ED_ to be 365.6 s^−1^ for the ^5^D_0_ → ^7^F_2_ transition and 123.1 s^−1^ for the ^5^D_0_ → ^7^F_4_ transition in [BMIM][PF_6_], using the *k*_R_ value of 287.2 s^−1^ obtained from Equation (3). The non-radiative rate constant (*k*_NR_) can be estimated using the relationship below:

(7) $$k_{\mathrm{NR}}=\frac{1}{\tau_{\mathrm{obs}}}-k_{R}.$$

In [BMIM][PF_6_], the *k*_NR_ value for the emissive ^5^D_0_ excited state is 388.5 s^−1^, which is comparable to that in acetonitrile. Thus, the effect of the solvent on the non-radiative processes of the Eu^3+^ ion is also equivalent between [BMIM][PF_6_] and acetonitrile.

Interestingly, the *η*_EnT_ value calculated using Equations (1) and (2) is 76.0% in [BMIM][PF_6_], which is significantly larger than that in acetonitrile (29.1%). It is well known that the energy transfer process in Ln complexes is significantly affected by the energy donor level and by the distance between the metal and ligand. In the present work, the latter effect may be negligible since the ligand field symmetry around the Eu^3+^ ion appears to be independent of the solvent, meaning that the electric structure of the ligand in [BMIM][PF_6_] may enhance the energy transfer efficiency in the IL compared to that in acetonitrile.

#### 2.1.3. Estimation of the Energy Donor State

The energy transfer efficiency greatly depends on the process by which the donor state is deactivated, as in the following equation:

(8) $$\eta_{\mathrm{EnT}}=\frac{k_{\mathrm{EnT}}}{k_{\mathrm{EnT}}+k_{R}(\mathrm{donor})+k_{\mathrm{NR}}(\mathrm{donor})}.$$

**Figure 4:**
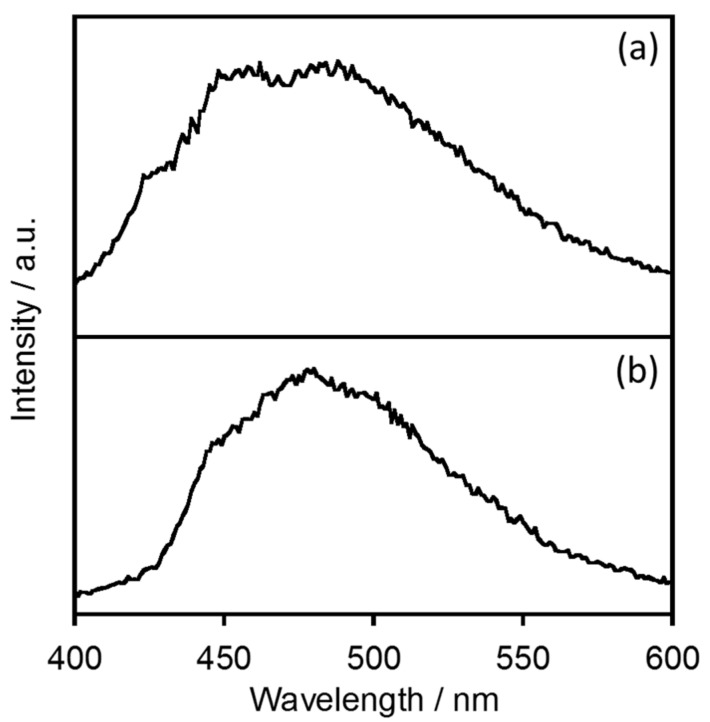
Phosphorescence spectra localized on the ligand moiety of helical Gd complex with L in (**a**) [BMIM][PF_6_] and (**b**) ethanol at 77 K (*λ*_ex_ = 328 nm).

In the case of EuL, the triplet state of the ligand functions as the energy donor to the Eu^3+^ ion [4]. The energy donor level in [BMIM][PF_6_] was estimated by acquiring phosphorescence spectra of a gadolinium complex with L (abbreviated as GdL) at 77 K (Figure 4). GdL generates a phosphorescence band at approximately 460 nm in both [BMIM][PF_6_] and the rigid solvent ethanol, as shown in Figure 4.

However, the phosphorescence lifetime (*τ*_T_) of GdL in [BMIM][PF_6_] (200 ns) was quite different from that in ethanol (5 ns, Figure S5 and Table 2). Since the phosphorescence quantum yield (*ϕ*_T_) of GdL is less than 0.1% in both solvents, the non-radiative rate constant of the triplet state (*k*_NR_(T)) can be directly approximated by *τ*_T_ as in the following equations.

(9) $$\phi_{T}=k_{R}(T)\times \tau_{T}=\frac{k_{R}(T)}{k_{R}(T)+k_{\mathrm{NR}}(T)}<0.00$$

(10) $$k_{R}(T)<<k_{\mathrm{NR}}(T)$$

(11) $$\tau_{T}=\frac{1}{k_{R}(T)+k_{\mathrm{NR}}(T)}\sim \frac{1}{k_{\mathrm{NR}}(T)}.$$

The *k*_NR_(T) value for GdL in [BMIM][PF_6_] is estimated to be 5.0 × 10^6^ s^−1^, which is much smaller than that in methanol (2.0 × 10^8^ s^−1^). Thus, the energy transfer from the ligand to the Eu^3+^ ion is accelerated in [BMIM][PF_6_] compared to that in organic solvents.

#### 2.1.4. Effect of the Viscosity of [BMIM][PF_6_] on the Energy Transfer Efficiency of EuL

**Figure 5:**
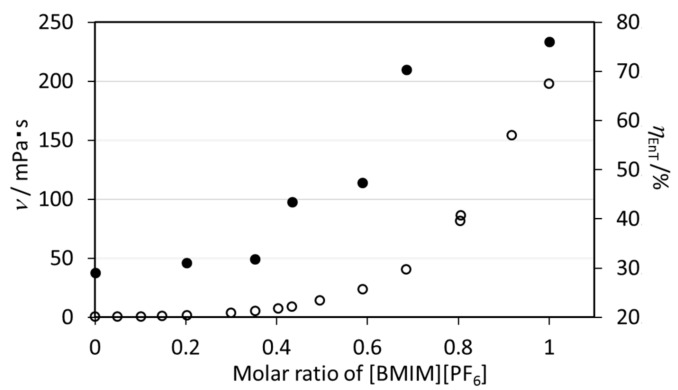
*η*_EnT_ (filled circles) and *ν* (open circles) as functions of the [BMIM][PF_6_] mole fraction.

It is well known that ILs have higher viscosities than organic solvents [26]. The viscosity of solvents may affect the molecular distortion or intermolecular interactions of the Eu complex, which in turn have significant effects on the nonradiative relaxation process, as discussed above. The impact of solvent viscosity on the energy transfer process in EuL was assessed by estimating the rate constants and efficiencies of the energy transfer pathway in mixtures of [BMIM][PF_6_] and acetonitrile with various proportions (Table 3, Figure S3) [27]. The kinetic viscosity of each mixture (*ν*) will vary with the molar ratio of [BMIM][PF_6_] to acetonitrile. However, the *τ*_obs_, *k*_R_, and *k*_NR_ values are independent of the kinetic viscosity, meaning that the ligand field of the Eu^3+^ ion is only minimally affected by viscosity. In contrast, the *η*_EnT_ value is drastically increased at higher viscosities, as shown in Figure 5. It is therefore evident that the more viscous [BMIM][PF_6_] suppresses the nonradiative relaxation of the ligand, as discussed in Section 2.1.2. The result is that the EuL exhibits higher emission efficiency in [BMIM][PF_6_] than in organic solvents.

### 2.2. Temperature Dependence of the Structure and Luminescence Properties of EuL in [EMIM][PF_6_]

#### 2.2.1. Luminescence Properties

**Figure 6:**
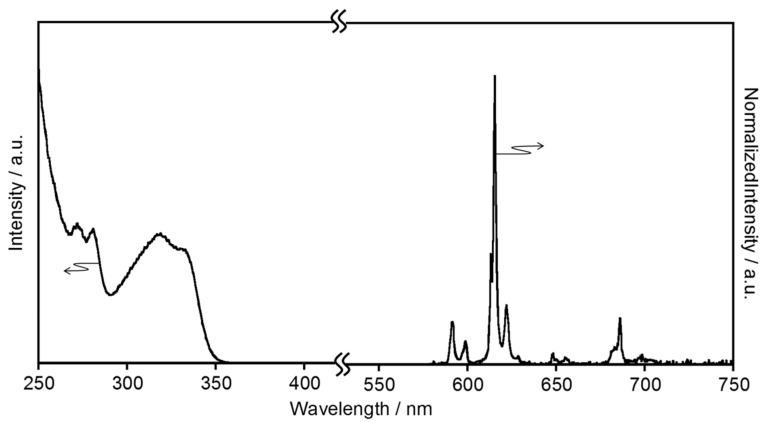
Excitation (left) and emission (right) spectra of EuL in [EMIM][PF_6_] (*λ*_mon_ = 616 nm, *λ*_ex_ = 328 nm).

[EMIM][PF_6_] is a solid at room temperature, as its melting point is around 60 °C. The emission and excitation spectra of EuL dispersed in [EMIM][PF_6_] are shown in Figure 6. In [EMIM][PF_6_], the luminescence bands are observed at the same position as in [BMIM][PF_6_] or acetonitrile. The excitation spectrum acquired in [EMIM][PF_6_] also corresponds to that in [BMIM][PF_6_]. These spectral results indicate that EuL dispersed in [EMIM][PF_6_] does not undergo intramolecular interactions. That is, the molecular structure of EuL is maintained in [EMIM][PF_6_] just as well as in [BMIM][PF_6_]. The *ϕ*_ff_ value for EuL in [EMIM][PF_6_] is, however, much larger than that in [BMIM][PF_6_] (Table 4) and comparable to that of solid EuL (52.6%) [4]. This increase in *ϕ*_ff_ results from efficient energy transfer from the ligand to the Eu^3+^ ion in [EMIM][PF_6_] (*η*_EnT_ > 99%), since [EMIM][PF_6_] works as a rigid medium and thus mimics the solid state.

#### 2.2.2. Structural Analyses

**Figure 7:**
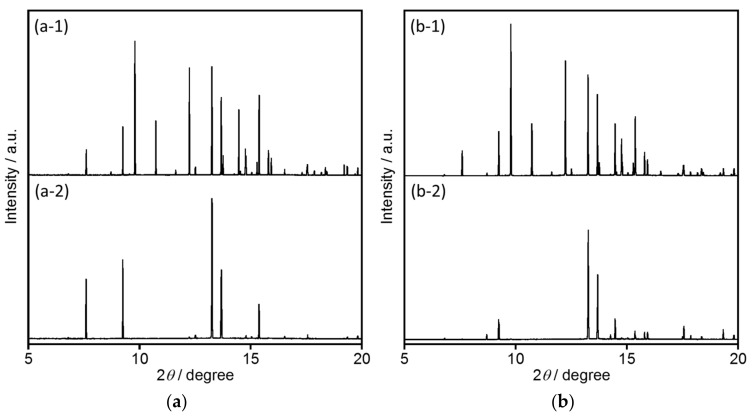
Synchrotron X-ray powder diffraction patterns of (**a**) [EMIM][PF_6_] and (**b**) EuL in [EMIM][PF_6_]. (**a-1**,**b-1**) before and (**a-2**,**b-2**) after heating at 70 °C (*λ* = 1.017593 Å).

The crystal structure of [EMIM][PF_6_] has already been reported [28] to be monoclinic (space group *P*2_1_/c). In this work, synchrotron X-ray powder diffraction (XRPD) analyses of [EMIM][PF_6_] were performed before and after heating, with the results shown in Figure 7a. Diffraction peaks are evident at 2*θ* values of 7.6, 9.2, 9.8, 10.8, 12.2, 13.3, 13.7, 13.8, 14.5, 14.7, 15.2, and 15.5° and can be assigned to the (0 1 1), (1 1 0), (1 0 −2), (0 1 2), (1 0 2), (0 0 3), (0 2 1), (2 0 0), (1 2 −2), (2 1 −1), (0 2 2), and (1 2 1) planes, respectively. After re-crystallization of [EMIM][PF_6_] following heating at 70 °C, certain diffraction peaks, such as those for the (1 0 −2), (0 1 2), and (1 0 2) planes, disappear. In these crystal planes (Figure S4a–c), EMIM and PF_6_^−^ molecules exist in an alternating arrangement. Thus, the disappearance of these crystal planes suggests that the [EMIM][PF_6_] is selectively orientated in the direction of the (0 1 1) and (1 1 0) planes (Figure S4d,e), such that EMIM molecules and PF_6_^−^ counter anions form separate layers. The diffraction pattern of [EMIM][PF_6_] prior to heating is affected by the addition of EuL (Figure 7b). In addition, following the re-crystallization of [EMIM][PF_6_] containing EuL, the same diffraction peaks disappear. Additionally, the presence of EuL eliminates the (0 1 1) and (1 1 0) diffractions of the [EMIM][PF_6_]. These results indicate that Eu^3+^ ions, having a high atomic scattering factor, are readily incorporated into the EMIM or PF_6_ layers in conjunction with the re-orientation of the [EMIM][PF_6_].

#### 2.2.3. Temperature Dependence of Luminescence Properties

**Figure 8:**
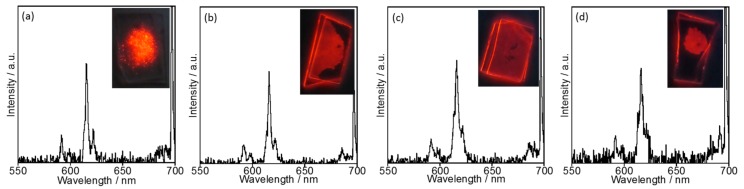
In situ temperature dependence of emission spectra with photographs of EuL in [EMIM][PF_6_] at (**a**) 17, (**b**) 61, (**c**) 108, and (**d**) 121 °C (*λ*_ex_ = 254 nm).

Interestingly, the bright red luminescence of EuL was maintained in [EMIM][PF_6_] even at 120 °C. Figure 8 presents the emission spectra of EuL in [EMIM][PF_6_] at various temperatures and demonstrates very little change in the spectra up to 120 °C. In contrast, in acetonitrile solution, the EuL was decomposed at temperatures above 100 °C such that the emission ceased. It therefore appears that EuL remains in a highly stable structure in [EMIM][PF_6_] even above 100 °C.

## 3. Materials and Methods

### 3.1. Reagents and Materials

The majority of reagents and solvents were used as-received, without further purification from Kanto Chemical Co. Inc. (Tokyo, Japan) and Wako Chemicals (Osaka, Japan). EuL ([Eu(L)(NO_3_)_2_](PF_6_)) was synthesized using a previously reported method [4]. Subsequently, the EuL (0.178 μmol) was dissolved in [BMIM][PF_6_] (24.2 mmol, approximately 5 mL) and stirred for 10 min at 80 °C to dissolve the complex. Solutions of EuL in [BMIM][PF_6_] and [EMIM][PF_6_] were clear under white light and showed red emission under UV light.

### 3.2. Measurements

Synchrotron XRPD data were acquired using the large Debye-Scherrer camera installed at the SPring-8 BL02B2 beamline, employing an imaging plate as a detector. The N_2_ gas flow system was also installed to allow for low-temperature experiments (down to 77 K).

Electronic absorption and luminescence spectra were recorded on a photo spectrometer UV-3100 Shimadzu (Kyoto, Japan) an absolute specular reflectance attachment and a fluorophoto spectrometer Fluorolog 3–22 Horiba Jobin-Ybon Ltd. (Kyoto, Japan), respectively. Emission decay curves were obtained using a Quantaurus-tau C11367-12 , Hamamatsu Photonics K. K. (Hamamatsu, Japan) with excitation via a xenon flash lamp with a band-path filter (λ_ex_ = 340 nm) and an LED light source for the measurement of ff emission and phosphorescence lifetimes, respectively. The fluorescence quantum yields were determined using a C9920-02 Absolute PL Quantum Yield Measurement System, Hamamatsu Photonics K. K. In situ luminescence spectra at high temperatures were recorded on USB2000 spectrometer equipped with an optical fiber, Ocean Optics Inc. (Dunedin, FL, USA) under UV light.

## 4. Conclusions

We demonstrated that the intramolecular energy transfer in a helical Eu complex (EuL) is accelerated in ILs, resulting in higher emission efficiencies relative to those obtained in organic solvents. The single helical structure of EuL is evidently maintained in both [BMIM][PF_6_] and [EMIM][PF_6_], just as in acetonitrile. EuL in [BMIM][PF_6_] generates bright red luminescence with a quantum yield of 32.3%. This value is significantly greater than that in acetonitrile (12%). Estimations of rate constants associated with energy relaxation show that the energy transfer process is more efficient in [BMIM][PF_6_]. This effect is attributed to the suppression of molecular fluctuations in the ligand. Both the helical structure and strong luminescence of EuL can be maintained in [EMIM][PF_6_] up to 120 °C. To the best of our knowledge, this is the first report of enhanced intramolecular energy transfer in a Eu complex following stabilization of the coordination structure in ILs. These data suggest that the luminescence of various other lanthanide complexes should also be enhanced in ILs.

## Figures and Tables

**Table 1:** Luminescence quantum yields (*ϕ*_ff_) and luminescence lifetimes (*τ*_obs_) of the helical Eu complex with L in [BMIM][PF_6_] and in acetonitrile [4] (^a^: *λ*_ex_ = 328 nm, *λ*_mon_ = 550–782 nm, ^b^: *λ*_ex_ = 340 nm, *λ*_mon_ = 616 nm).

	*ϕ*_ff_ (%) ^a^	*τ*_obs_ (ms) ^b^
in [BMIM][PF_6_]	32.3	1.48
in acetonitrile	12.0	1.55

**Table 2:** *ϕ*_T_, *τ*_T_, and *k*_NR_ for the triplet state (*k*_NR_(T)) of the ligand L of helical Gd complex as GdL in [BMIM][PF_6_] and in ethanol at 77 K (^a^: *λ*_ex_ = 328 nm, *λ*_mon_ = 400–600 nm, ^b^: *λ*_ex_ = 340 nm, *λ*_mon_ = 488 nm).

	*ϕ*_T_ (%) ^a^	*τ*_T_(ns) ^b^	*k_NR_*(T) (s^−1^)
in [BMIM][PF_6_]	<0.1	ca. 200	5.0 × 10^6^
in ethanol	<0.1	ca. 5	2.0 × 10^8^

**Table 3:** *ϕ*_ff_, *τ*_obs_, *n*, *k*_R_, *k*_NR_, and *η*_EnT_ values for EuL in mixed solvents of [BMIM][PF_6_] and acetonitrile along with the kinetic viscosity values (*ν*) [27] (^a^: *λ*_ex_ = 328 nm, *λ*_mon_ = 550–780 nm, ^b^: *λ*_ex_ = 340 nm, *λ*_mon_ = 616 nm).

Molar Ratios [BMIM][PF_6_]: Acetonitrile		*ν* (mPa·s)	*ϕ*_ff_(%) ^a^	*τ*_obs_(ms) ^b^	*k*_R_ (s^−1^)	*k*_NR_ (s^−1^)	*η*_EnT_ (%)
1.00	0.00	198.18	32.3	1.46	287.2	388.5	76.0
0.64	0.36	40.69	30.0	1.42	300.7	403.6	70.3
0.59	0.41	23.84	17.6	1.46	254.3	430.6	47.4
0.43	0.75	9.15	15.8	1.43	254.6	444.7	43.4
0.35	0.65	5.31	12.1	1.49	255.2	416.0	31.8
0.20	0.80	1.87	11.6	1.45	257.6	432.0	31.1
0.00	1.00	0.48	12.0	1.55	266.2	379.0	29.1

**Table 4:** *ϕ*_ff_, *τ*_obs_, and *η*_EnT_ values of EuL in [EMIM][PF_6_] (^a^: *λ*_ex_ = 328 nm, *λ*_mon_ = 550–782 nm, ^b^: *λ*_ex_ = 340 nm, *λ*_mon_ = 616 nm).

*ϕ*_ff_ (%) ^a^	*τ*_obs_ (ms) ^b^	*η*_EnT_ (%)
52.1	1.37	>99

## Supplementary Materials

The following are available online. Figure S1: Luminescence decay curves of EuL in [BMIM][PF_6_] and acetonitrile; Figure S2: Excitation spectra of EuL in [BMIM][PF_6_] and in acetonitrile; Figure S3: Electronic absorption, emission spectra, and decay curves of EuL in [BMIM][PF_6_]/acetonitrile; Figure S4: Projection views of a [EMIM][PF_6_] single crystal.
