# Supplementary material for: The Enhanced Intramolecular Energy Transfer and Strengthened ff Luminescence of a Stable Helical Eu Complex in Ionic Liquids
Source: Molecules. 2018 Jan 24;23(2):55. doi: 10.3390/molecules23020055 (PMC6017298; doi:10.3390/molecules23020055)
Supplement: Supplementary file 1 [file molecules-23-00055-s001.pdf]

Supporting Information

Enhanced Intramolecular Energy Transfer with Strengthen ff Luminescence of a Stable Helical Eu Complex in ionic liquid

Yuki Hasegawa [1], Ayumi Ishii [1,2] *, Yudai Inazuka [1], Naho Yajima [1], Shogo Kawaguchi [3], Kunihisa Sugimoto [3], and Miki Hasegawa [1,*]

1 Department of Chemistry and Biological Science, College of Science and Engineering, Aoyama Gakuin University;
2 JST, PRESTO, 4-1-8 Honcho, Kawaguchi, Saitama, 332-0012, Japan
3 Research & Utilization Division, Japan Synchrotron Radiation Research Institute (JASRI/SPring-8), 1-1-1, Kouto, Sayo, Hyogo, 679-5198, Japan
* Correspondence: hasemiki@chem.aoyama.ac.jp & ayumi@chem.aoyama.ac.jp; Tel.: +81-42-759-6221;

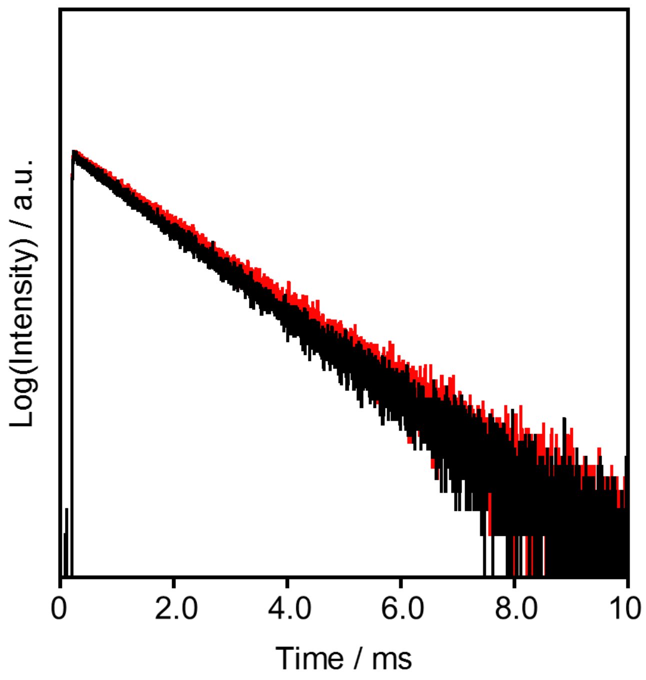

Figure S1. Luminescence decay curves of EuL in [BMIM][PF_6] (black line) and acetonitrile (red line) (λ_{ex} = 340 nm, λ_{mon} = 616 nm).

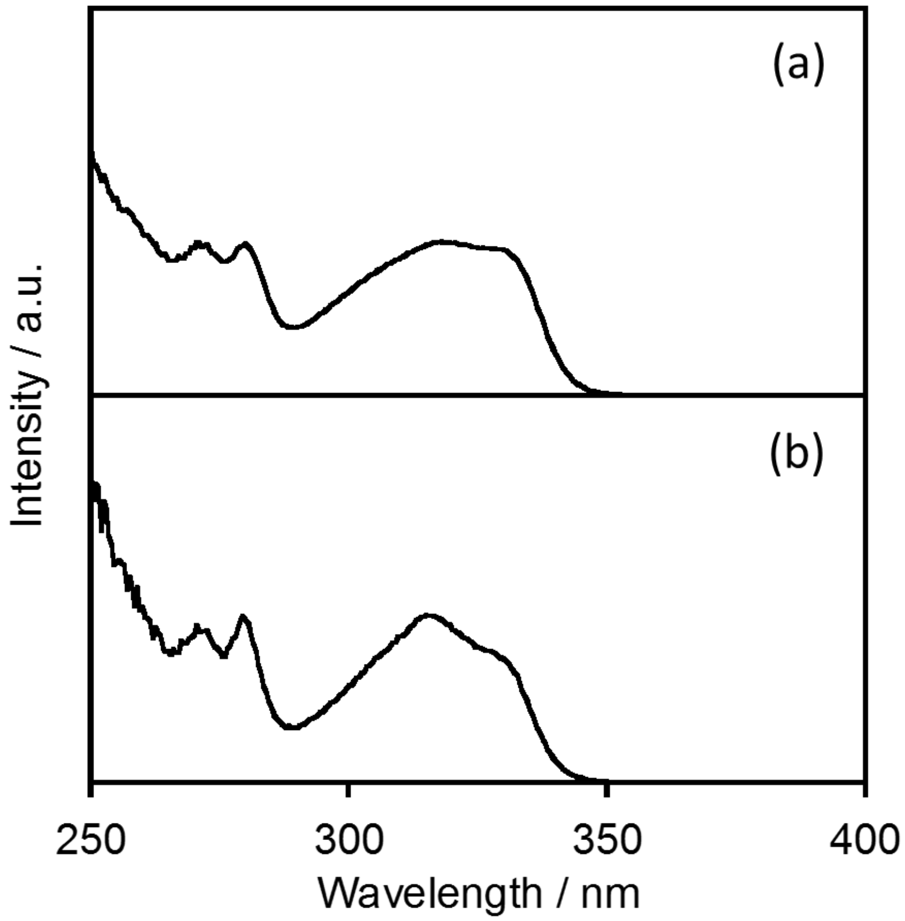

Figure S2. The excitation spectra of Eu**L** (a) in [BMIM][PF_6] and (b) in acetonitrile monitored at 616 nm.

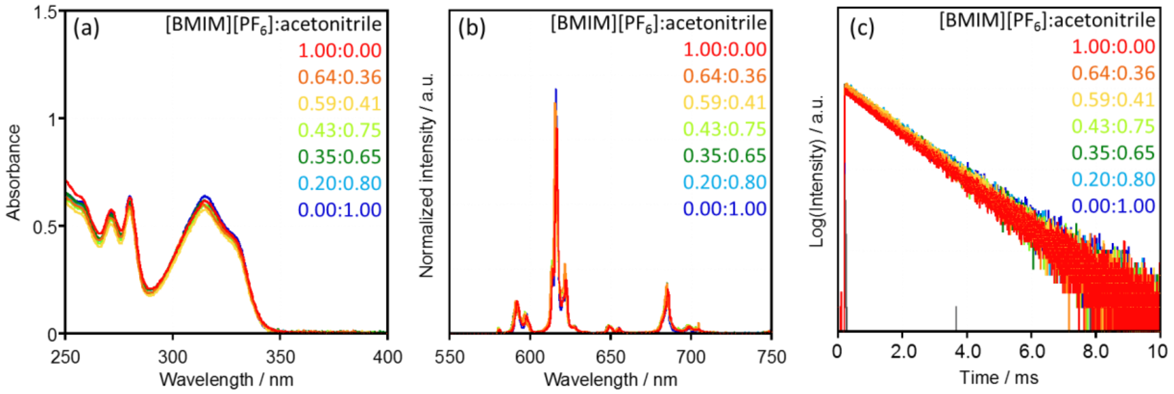

Figure S3. (a) Electronic absorption, (b) emission spectra (λ_{ex} = 328 nm) and (c) decay curves (λ_{ex} = 340 nm, λ_{mon} = 616 nm) of Eu**L** in [BMIM][PF_6]/acetonitrile with various concentrations.

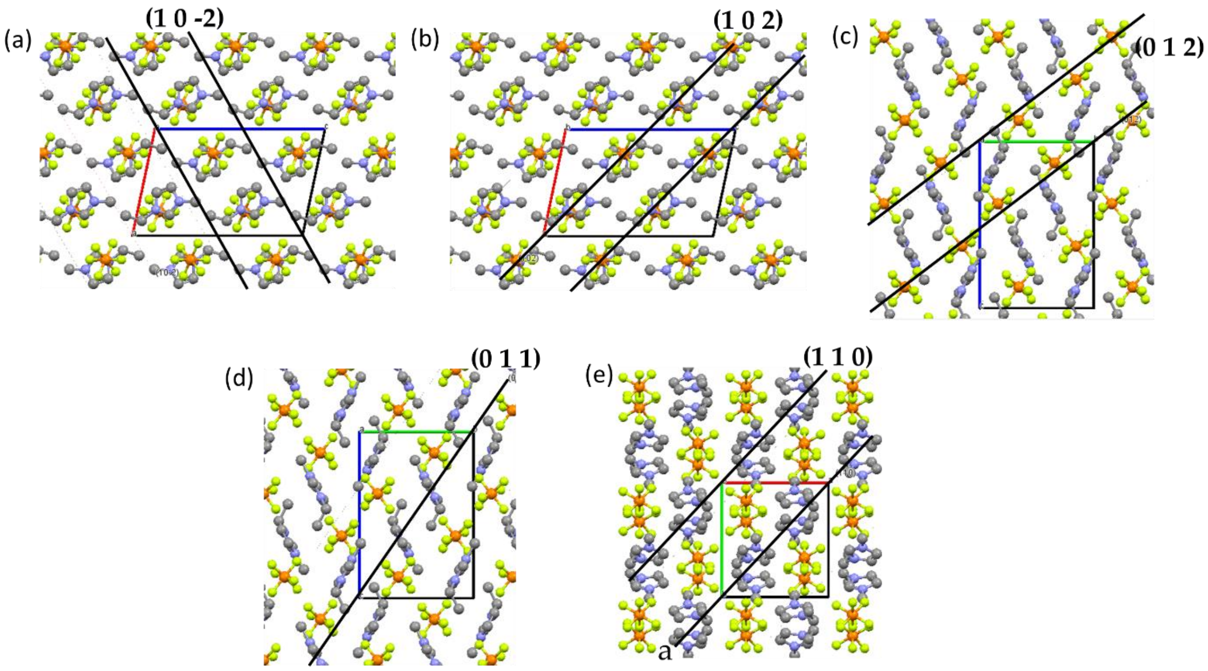

Figure S4. Projection views of the single crystal of [EMIM][PF_6] along with (a) (10-2), (b) (102), (c) (012), (d) (011), and (e) (110) planes
.

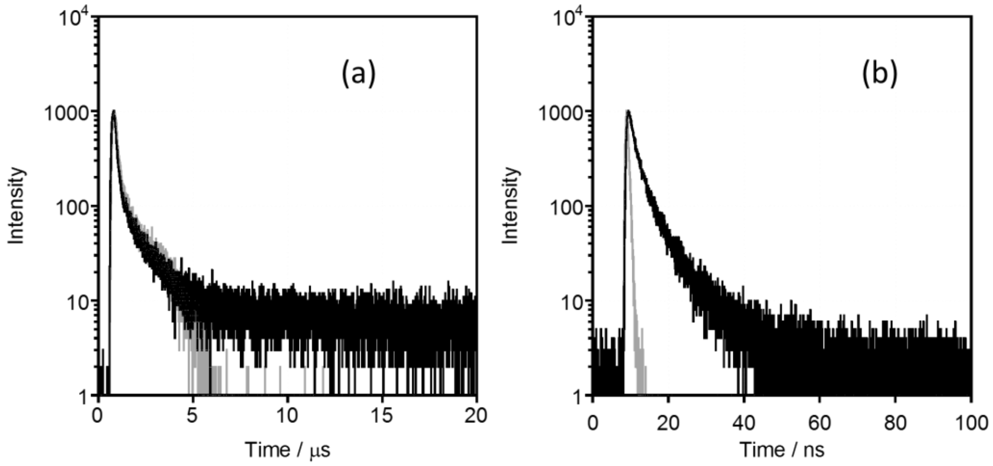

Figure S5. Phosphorescence decay curves (black line) localized on the ligand of GdL at 77 K (a) in [BMIM][PF_6] and (b) in ethanol. Grey line is IRF. (λ_{ex} = 340 nm, λ_{mon} = 488 nm)
